# Vladimir Sertić: forgotten pioneer of virology and bacteriophage therapy

**DOI:** 10.1098/rsnr.2019.0010

**Published:** 2020-01-08

**Authors:** Zdravko Lacković, Karlo Toljan

**Affiliations:** University of Zagreb School of Medicine, Šalata 11, Zagreb 10000, Croatia

**Keywords:** bacteriophages, bacteriophage φX174, phage therapy, Croatia

## Abstract

Vladimir Sertić was a pioneer of bacteriophage research in the period between the two world wars. He was born and educated in Croatia, where he made his initial discoveries, and joined Félix d'Herelle's Laboratoire du Bactériophage in Paris in 1928. Original documents and a box with hundreds of sealed bacteriophages samples were kept in Sertić's Zagreb home for decades. Following Vladimir's death, his sister passed this archival material to Professor Zdravko Lacković in 1989. Some years later, these artefacts were opened and studied. Additionally, we conducted a literature search using the term ‘Vladimir Sertić’ in the databases PubMed and Google Scholar. After a detailed examination of these data, we established a chronology of his work and compiled a list of his scientific publications. A complete bibliography, with the exception of those publications already cited here, is provided as an appendix. Sertić's key contributions included the exploration of the properties of phage lysins, the devising of a uniform bacteriophage classification system and, in collaboration with his protégé, Nikolai Boulgakov, the isolation of numerous bacteriophage strains, including the famous φX174. Finally it was Sertić's pioneering work in Zagreb that offered confirmation that phages are live agents.

## Introduction

**Figure 1.**
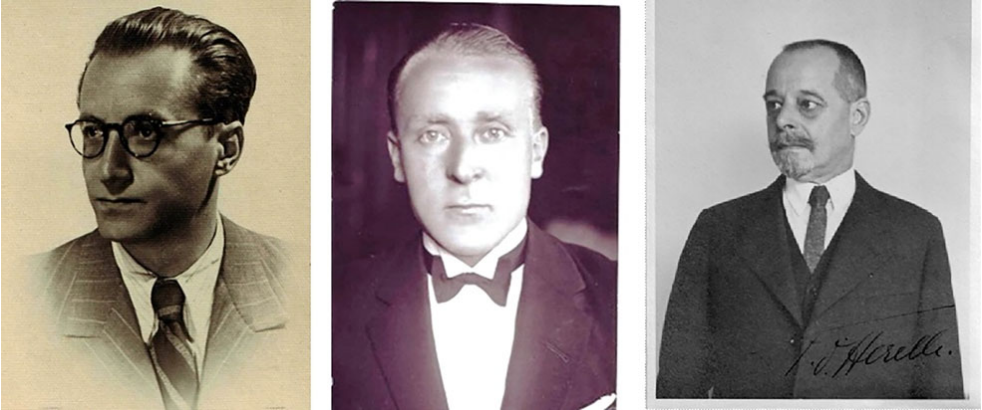
From left to right: Vladimir Sertić, Nikolai Boulgakov, Félix d'Herelle. An autograph by d'Herelle is seen on the photo, which was probably given as a souvenir. (From Sertić's family papers, currently kept by Z. Lacković.) (Online version in colour.)

Because of the growing problem of antibiotic resistance of bacteria, there has been a recent revival of interest in bacteriophage therapy.^1^ This emerging interest in those bacterial viruses in the biomedical community has brought the topic to a wider professional and general public audience. Whether one takes Frederick William Twort's first inconclusive finding published in 1915 or the more precise and insightful publication in 1917 by Félix Hubert d'Herelle to be the exact ‘birthdate’,^2^ phage research has a century-old history.^3^ Although the research and lives of d'Herelle and the Georgian scientist George Eliava are described and known,^4^ the two other important pioneers of bacteriophage research, Nikolai Boulgakov and especially Vladimir Sertić, have remained practically anonymous (figure 1).

Vladimir Sertić was a Croatian microbiologist who in the later phase of his career held the position of scientific director at d'Herelle's private institute in Paris, the Laboratoire du Bactériophage, and whose scientific contribution to research and application of phage therapy and lysins was only partially described in Croatian-language publications.^5^ Boulgakov (also spelled as Bulgakov or Buljgakov), the younger brother of the famous writer Mikhail, had left his homeland in Ukraine in 1922 because of the communist revolution, and settled in Zagreb. He took Yugoslavian citizenship and continued with his medical studies at the School of Medicine.^6^ Upon graduation, Nikolai joined Sertić as an assistant, and eventually followed him to Paris. Together they co-authored 20 papers, including those mentioning the famous φX174 virus particle. Apparently, it was first isolated and named by Sertić and Boulgakov in their own comprehensive and original bacteriophage classification.^7^ Moreover, Sertić himself was one of the first investigators to focus heavily on the properties and nature of phage (bacterio)lysins: bacteriolytic enzymes produced by phages. His findings were the first to correctly show that lysins were actual enzymes, produced and encoded by biologically active phages.^8^ These results were also in line with d'Herelle's claim that phages were, indeed, live agents.^9^

**Figure 2.**
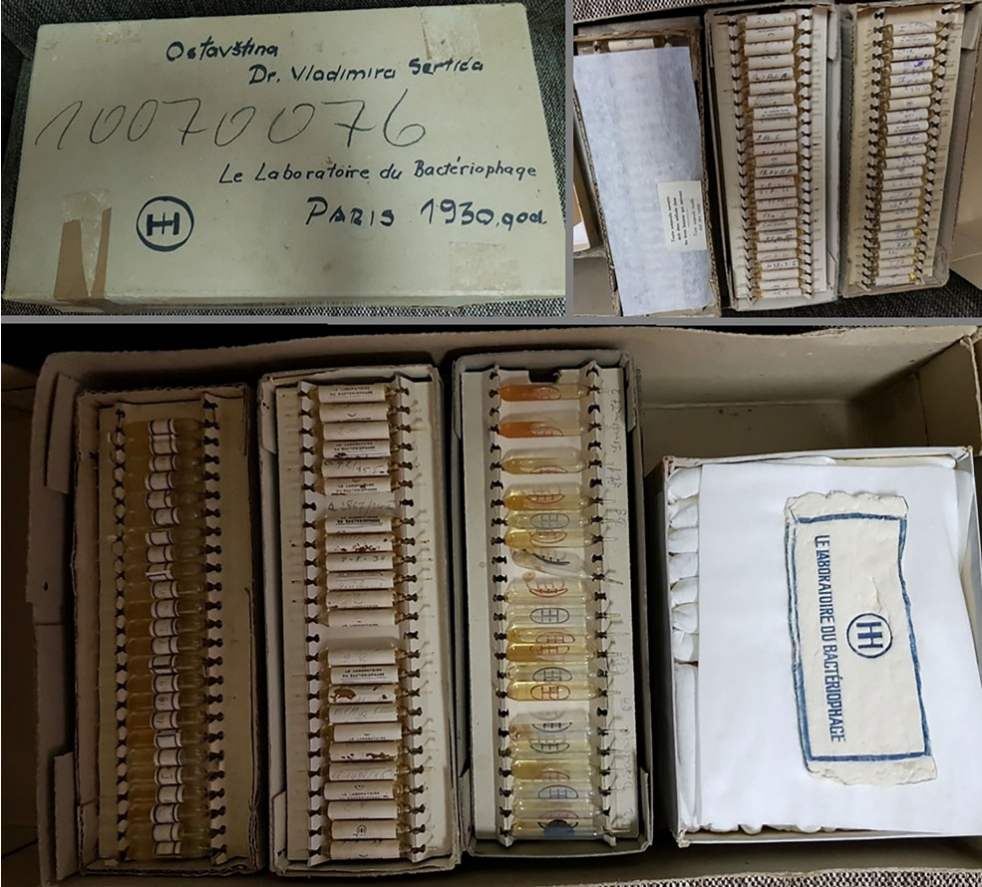
Recovered box containing multiple original therapeutic bacteriophage packages. (From Sertić's private archive.) (Online version in colour.)

**Figure 3.**
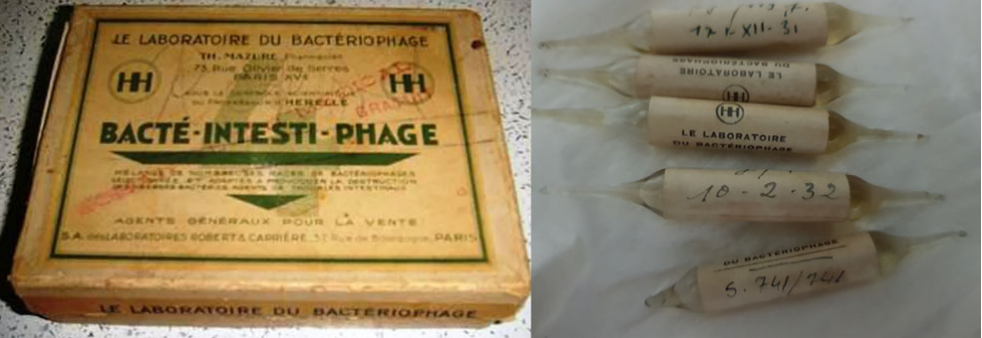
Phage preparations from d'Herelle's laboratory, indicating a widespread commercial presence. (From Sertić's private archive.) (Online version in colour.)

This story of their work would not have been uncovered had there not been an effort made by one of the authors (Lacković) from the Department of Pharmacology at the University of Zagreb School of Medicine. When preparing a manuscript in the late 1980s on scientific citation metrics from the affiliates of the School of Medicine before World War II, he encountered an unfamiliar name—that of Vladimir Sertić.^10^ At that time, it was barely possible to find anyone even recognizing the name of the late Professor Sertić, let alone able to provide any details about his life and work. Eventually, Lacković was able to locate his sister, Dr Mira Sertić, who was willing to make her brother's papers and records available. Among these private documents were multiple handwritten manuscripts, and final printed publications. Most exciting was a box dated 1930, packed with 520 authentic commercial ampoules containing phages produced in d'Herelle's laboratory, with 157 additional agar-filled vials, each with a culture of a named specific strain of bacteria—probably a reference culture collection for research purposes (see figures 2 and 3).

## A brief biography

Based on Sertić's archival material and through personal communication with his sister, it was possible to reconstruct his biography. He was born on 27 September 1901 in Gorica (Istria), then part of the Austro-Hungarian empire. Owing to his father's post as a senior army doctor in the Austro-Hungarian army, he spent his childhood and youth in various locations. Sertić opted to follow his father's career and, in 1919, became a student at the recently formed University of Zagreb School of Medicine. During his student days, he volunteered as a physician assistant at a military hospital in Zagreb, where his father was working. Assigned to bacteriology and venereal diseases wards, he was exposed to the evolving field of immunology and infectious diseases from his early student days. Thus, by the time of his graduation from medical school in 1925, he already had considerable laboratory experience.

After a prolific career as a researcher in Paris, Sertić returned to Zagreb in 1939, where, because of the outbreak of World War II, he accepted a post at the university's School of Medicine. Unfortunately, in 1943, just two years into his appointment as a professor in the Department of Bacteriology, Serology and Immunology, he was struck with a serious combination of pneumonia, heart failure and rheumatism. Ultimately, he suffered an unidentified psychiatric episode and, although he recovered his mental health during the following year, he was still afflicted with disability in the form of rheumatism and chronic heart failure, and was obliged to retire. Following the installation of a communist government in Yugoslavia in 1945, his retirement continued, with an extra disability support to supplement his meagre income. He lived with his sister in Zagreb. He died on 10 March 1983, a totally anonymous person to the research community of Croatia. By the end of the 1980s, nobody at the School of Medicine even remembered his name. His name was rediscovered only during a scientometric investigation by Lacković into the history of the School of Medicine in the late 1980s.^11^ It turned out that Sertić started his career in Lacković's own department—the Department of Pharmacology, then part of the Institute for Pharmacology and Experimental Pathology (Pathophysiology).

## Initial work in Zagreb

After completing his medical studies and internship at the Zagreb School of Medicine, Sertić briefly worked for the Ministry of Health. He obtained an academic position as a research fellow at the Institute for Pharmacology and Experimental Pathology (Pathophysiology) in 1926. Miroslav Mikuličić (Mikulichich, Miculichich), the head of the institute and a pupil of the Nobel laureate Otto Loewi,^12^ entrusted Sertić with the task of setting up an immunobiological ward. It was Mikuličić's idea to support a cross-disciplinary approach at the institute, where basic science and clinical wards were to be interconnected.^13^ In this setting, Sertić had the opportunity to pursue his interest in microbiology, which led him to his *in vitro* experiments with bacteriophages, by then a controversial topic with such prominent opponents to d'Herelle's ideas as Jules Bordet.^14^

**Figure 4.**
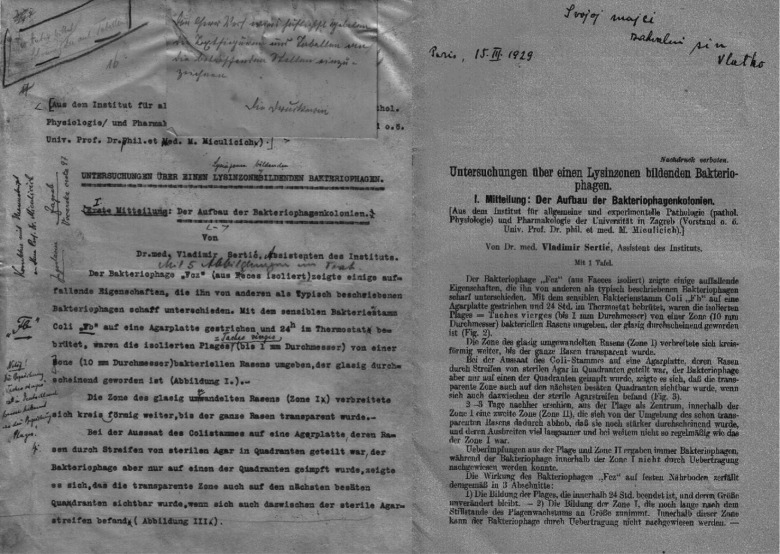
Sertić's initial paper from his experiments done in Zagreb; on the left is a draft copy with the author's remarks and corrections. (From Sertić's private archive.)

It was in these circumstances that Sertić published a paper in 1929 in which he described his experimental work done in Zagreb (figure 4).^15^ In it, he explained the occurrence of bacteriolysins (alternatively labelled as phage lysins, endolysins or simply lysins): bacteriolytic enzymes produced by phages. Initial speculations on lysins being specific phage-secreted bacteriolytic enzymes and not degradation-associated bacterial by-products were published by d'Herelle and Eliava as early as 1921.^16^ However, it was Sertić's pioneering work in Zagreb that offered final confirmation and validation of this postulate. This work also supported d'Herelle's claim that the original bacteriolytic effects that he observed were driven by viruses and their activity, and not by latent intrabacterial enzymes or other non-living factors, as proposed by his critics. In the 1929 paper, Sertić used phage based on *Escherichia coli* bacteria as a model. Even decades later, other investigators working on the topic of lysins still cited Sertić's ground-breaking paper as the one that brought the verdict on the nature of phage lysins, as well as discerning their respective bacteriolytic properties.^17^

## At d’Herelle's Parisian institute

Sertić was appointed as a scientific director at d'Herelle's private Parisian institute, the Laboratoire du Bactériophage, on 1 November 1928. He would hold that position until 1939 and the outbreak of World War II. His most prolific year was 1935, when he published 10 papers, mostly co-authored with Boulgakov, among which are the ones describing a unique phage classification system.^18^ One of the main drawbacks of phage research and therapy was the inconsistent naming of particular phage types, and this endeavour by Sertić and Boulgakov was an attempt to bring order to chaos. They even tested phage types from other or new sources, such as the public waste waters,^19^ and matched them to their phage database based on their developing classification principles.^20^ As with developments in the area of transfusion medicine,^21^ a uniform phage classification at the time represented a prevailing standard while also establishing the authority of d'Herelle's lab. Although a contemporary classification of viruses, including bacteriophages, has been developed by the International Committee on Taxonomy of Viruses (ICTV), original names assigned by Sertić are still used for many phage isolates; the best known example is φX174.^22^

**Figure 5.**
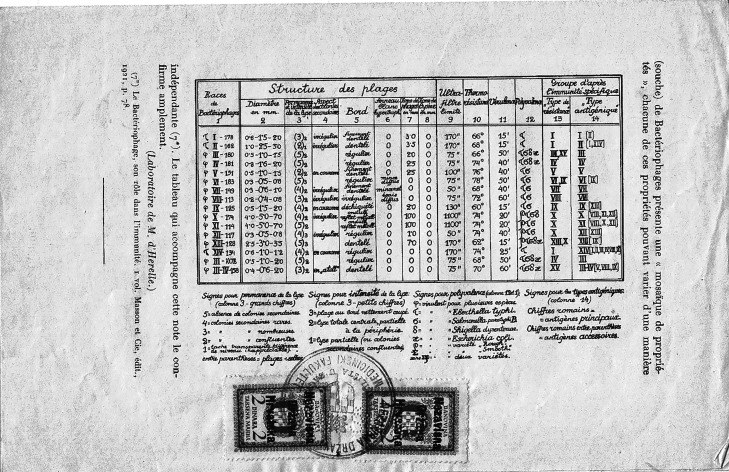
Bacteriophage classification from original 1935 publication by Sertić and Boulgakov. Note initial characterization of φX174. (From Vladimir Sertić and Nikolai Boulgakov, ‘Le groupement des bactériophages d'après leur type antigénique’, *C. R. Soc. Biol.* **119**, 983–985 (1935).)

Since the institute was privately run and commercially strong, Sertić's isolates reached many research centres, and the names of strains were kept in use in accordance with his classification. One exemplary phage is φX174, first mentioned by Sertić and Boulgakov when describing their classification system.^23^ The classification comprises a Greek letter, a Roman numeral and an Arabic number. The Greek letter φ was used if the phage acted against multiple bacteria, whereas other Greek letters (τ, σ, δ, etc.) were used if the phage was specific to a single bacterium species (figure 5). The Roman numerals comprised 14 consecutive numbers, I–XIV. Thus, the correct reading of φX174 is *phi ten* 174 and not *phi ex* 174. Roman numerals were used to describe ‘antigens’ carried by the phage. These ‘antigenic’ reactions were based on the then already known methodology, but in this case specifically applied by Sertić and Boulgakov, according to which ‘anti-sera’ were produced by injecting a pure line culture of a given phage isolate into rabbits.^24^ The rabbit serum was then used for cross-neutralization tests with other isolates; the neutralization by a specific serum was one of several properties that they used to classify phages into groups designated by Roman numerals. The Arabic number was given in accordance to isolates from the private phage collection of d'Herelle's laboratory, and was therefore an arbitrary component.

Sertić and Boulgakov's phage φX174 stands out for a number of reasons. Its DNA genome, the first example of a single-strand DNA as well as a covalent circular DNA, was the first one to be fully sequenced by Frederick Sanger's team, an achievement that garnered Sanger his second Nobel Prize, in 1980.^25^ Ten years earlier, the 1959 Nobel laureate Arthur Kornberg successfully managed to synthesize *in vitro* a biologically active, exact copy of φX174 DNA, thus setting a milestone in the field of synthetic biology.^26^ More recently, Craig Venter's group successfully assembled a strand of φX174 DNA by a full genome assembly method from synthetic oligonucleotides.^27^ Notably, for every modern experiment in which φX174 has been used, it is possible to trace a direct line back to the initial encounter in 1950s between Boulgakov and Robert Sinsheimer, when the latter obtained the virus, originally isolated and named by Sertić and Boulgakov.^28^ As a scientist located in USA, Sinsheimer spread the virus throughout that country's laboratories and thence worldwide, reaching investigators such as Sanger, working in the UK. It is not an overstatement to claim that there is a bit of Sertić and Boulgakov's legacy knitted into all these famous experiments and into current work, where φX174 serves as a common model. When using the search terms ‘φX174’ and ‘phi X 174’ as descriptors in the PubMed database, we found 1859 publications (retrieved 14 July 2017); there were 267 000 hits in the less selective and less specific Google Scholar database.

Sertić did not lose focus on his main duties of developing phage therapy, and started tackling the topic of bacteriolysins in more detail.^29^ At the time, lysins were seen as a perfect tool for diagnosing specific bacterial strains. He and Boulgakov continued to perfect the isolation of various lysins and found them to be highly specific and sensitive for a specific strain, an improvement over any other method of detection at the time. After his initial 1929 paper, Sertić published eight more papers based on phage lysins. They include his final three publications (two co-authored with Boulgakov), printed in 1939, on the eve of World War II.

## Demise of d’Herelle's institute

All of Europe was turned into a battlefield with the advent of World War II. Boulgakov, being a Yugoslav citizen, spent time as a prisoner in the Nazi internment camp near Campiègne in northern France.^30^ During the summer of 1939, Sertić took leave to spend time in Croatia. As a result of wartime exigencies in the Balkans, he was mobilized by the Yugoslav army in the spring of 1941, was briefly held captive and was then released by the Nazi occupiers back to the newly formed Independent State of Croatia. He was appointed as a professor and later Head of the Department for Bacteriology at the Zagreb School of Medicine in the autumn of 1941. From that period a handwritten and partially typed manuscript of what was intended to be a microbiology textbook has been retrieved from Sertić's papers. It consists of roughly 300 pages, describing the most up-to-date knowledge in bacteriology, virology and immunology, with almost a third of the manuscript being dedicated to the topic of bacteriophage.

In the events that brought chaos to Europe, d'Herelle's laboratory ceased to exist and, as others have documented, phage therapy essentially disappeared in the Western world. With antibiotics becoming the standard of care for treating infections, bacteriophages faded quickly from clinical practice only to blossom again as molecular biologists embraced phages as the central model for the gene; simple genomes, such as that of φX174, were crucial to much of the progress in basic research in the second half of the twentieth century.

## Sertić's legacy: phage and lysin therapy today

Evolving antibiotic resistance is almost universally viewed as an impending global health crisis.^31^ By applying phages targeted specifically to individual patient isolates—the most common of which are *E. coli* and *Pseudomonas aeruginosa—*a better clinical outcome may be possible. Using the PubMed database as a source, we found a slow resurgence in the number of publications on therapeutic phages since the beginning of this century: in the year 2000 the total number of papers was 1050; this had risen to 1380 by 2016, with a yearly trend for a few of those being classified as clinical trials. Additionally, lysins have emerged as potential alternatives to antibiotics. During the second half of the twentieth century, some commercial kits for rapid bacteria detection contained lysins as part of the approach to enhance specificity.^32^ Currently, lysins are seen as tools that may yield effective bactericidal therapies, and the scientific interest is considerable. A search on 25 October 2017, using the term ‘lysin’, returned 2256 publications in the PubMed database, 578 of which were published in or after 2011.

Based on Sertić's scientific bibliography, we can conclude that he was a prolific contributor to the phage research in the period before World War II. He published 35 papers in a ten-year period, most of those while working in d'Herelle's laboratory as the key person for research, development and devising of bacteriophage therapies destined for commercial use. A comprehensive bibliography of these publications, with the exception of those publications already cited in this paper, is provided in the appendix. Unfortunately, no peer-reviewed reports on actual therapeutic efficacy from that time are available. However, with the recent reconsiderations of phage or lysin therapy as a life-saving option when insurmountable antibiotic resistance is encountered, the role of the pioneers of bacteriophage research, including Vladimir Sertić, should not be forgotten.

## Footnotes

1 See, for example, William C. Summers, ‘Bacteriophage therapy’, *Ann. Rev. Microbiol.* **55**, 437–451 (2001).

2 Félix d'Herelle, ‘Sur un microbe invisible antagoniste des bacilles dysentériques’, *Comptes Rendus de l'Académie des Sciences* **165**, 371–375 (1917).

3 Donna H. Duckworth, ‘Who discovered bacteriophage?’, *Bacteriol. Rev.* **40**(4), 739–802 (1976); William C. Summers, ‘The strange history of phage therapy’, *Bacteriophage* **2**(2), 130–133 (2012).

4 I. A. Georgadze and E. G. Makashvili, ‘George Eliava’, in *The Georgian Soviet encyclopedia*, vol. 4 (Tbilisi, 1979), p. 5; William C. Summers, *Felix d'Herelle and the origins of molecular biology* (Yale University Press, New Haven, 1999).

5 Zdravko Lacković, ‘Katedra za Farmakologiju’ [‘Department of Pharmacology’], in *Medicinski Fakultet Sveučilišta u Zagrebu* [*University of Zagreb School of Medicine*], (ed. L. J. Čečuk, B. Belitza and M. Škrbić) (Stvarnost, Zagreb, 1984), pp. 369–373; Lacković Z, Čečuk Lj, Buneta Z. Mjera za znanost [Measure of Science]. (Medicinska Naklada, Zagreb, 1991); Marijan Klarica and Zdravko Lacković, ‘Prikaz rada djelatnika Zavoda za Farmakologiju između 90. i 95. obljetnice zavoda i dvije neobične priče iz zavodske povijesti’ [‘Presentation of the work of the Pharmacology Institute staff between the 90th and 95th anniversaries of the institute and two unusual stories from the institute's history’], *Mef.hr* **33**(2), 87–89 (2014).

6 Lesley Milne (ed.), *Bulgakov: the novelist-playwright* (Taylor & Francis e-Library, Amsterdam, 2005).

7 Vladimir Sertić and Nikolai Boulgakov, ‘Le groupement des bactériophages d'après leur type antigénique’, *C. R. Soc. Biol.* **119**, 983–985 (1935); ‘Le groupement des bactériophages suivant le type de résistance bactérienne’, *C. R. Soc. Biol.* **119**, 985–987 (1935).

8 Vladimir Sertić, ‘Untersuchungen über einen Lysinzonen bildenden Bakteriofagen’, *Zbl. Bakteriol. Parasit.* **110**, 125–139 (1929).

9 D'Herelle, *op. cit*. (note 2).

10 Zdravko Lacković, Anamarija Bekavac and Ljubomir Čečuk, ‘Croatian medical science from the foundation of the School of Medicine until 1941’, *Croat. Med. J.* **1**(1), 45–63 (1992).

11 *Ibid*.

12 Vladimir Dugački, ‘Prof. Dr. Phil. et Med. Miroslav Mikuličić, “enfant terrible” Zagrebačkoga Medicinskog Fakulteta’, *Mef.hr* **28**(1–2), 87–89 (2008).

13 Lacković, *op. cit*. (note 5); Dugački, *op. cit*. (note 12).

14 Emiliano Fruciano and Shawna Bourne, ‘Phage as an antimicrobial agent: d'Herelle's heretical theories and their role in the decline of phage prophylaxis in the West’, *Can. J. Infect. Dis. Med.* **18**(1), 19–26 (2007).

15 Sertić, *op. cit*. (note 8).

16 Felix d'Herelle and Georges Eliava, ‘Unicité du bacteriophage: sur la lysine du bactériophage’, *C. R. Soc. Biol.* **85**, 701–702 (1921).

17 Alice C. Evans, ‘The potency of nascent streptococcus bacteriophage B’, *J. Bacteriol.* **39**(5), 597–604 (1940): James C. Humphries, ‘Enzymic activity of bacteriophage-culture lysates 1: a capsule lysin active against *Klebsiella pneumoniae* type A1’, *J. Bacteriol.* **56**(5), 683–693 (1948).

18 Vladimir Sertić and Nikolai Boulgakov, ‘Classification et identification des typhi-phages’, *C. R. Soc. Biol.* **119**, 1270–1272 (1935); Sertić and Boulgakov, *op. cit*. (note 7).

19 Nikolai Boulgakov and Vladimir Sertić, ‘Sur des races de staphylophages isolées de l'eau d’égout’, *C. R. Soc. Biol.* **104**, 1258–1260 (1930).

20 Vladimir Sertić and Nikolai Boulgakov, ‘Technique de l'isolement de bactériophages en lignées pures’, *C. R. Soc. Biol.* **119**, 690–692 (1935); ‘Technique de l'identification des races de bactériophages’, *C. R. Soc. Biol.* **119**, 826–828 (1935).

21 Jenny Bangham, ‘Writing, printing, speaking: rhesus blood-group genetics and nomenclatures in the mid-twentieth century’, *Brit. J. Hist. Sci.* **47**(2), 335–361 (2014).

22 See ICTV, ‘Taxonomy’, https://Talk.Ictvonline.Org/Taxonomy/ (accessed 25 October 2017).

23 Sertić and Boulgakov, *op. cit*. (note 7); Sertić and Boulgakov, *op. cit*. (note 18).

24 Sertić and Boulgakov, *op. cit*. (note 20).

25 Frederick Sanger *et al.*, ‘Nucleotide sequence of bacteriophage φX174 DNA’, *Nature* **265**, 687–695 (1977). Sanger had received his first Nobel Prize in 1958 for his work on protein structure, especially that of insulin.

26 Mehran Goulian, Arthur Kornberg and Robert L. Sinsheimer, ‘Enzymatic synthesis of DNA, XXIV: synthesis of infectious phage phi-X174 DNA’, *Proc. Natl Acad. Sci. USA* **58**(6), 2321–2328 (1967).

27 Hamilton O. Smith *et al.*, ‘Generating a synthetic genome by whole genome assembly: φX174 bacteriophage from synthetic oligonucleotides’, *Proc. Natl Acad. Sci. USA* **100**(26), 15440–15445 (2003).

28 Robert L. Sinsheimer, ‘Purification and properties of bacteriophage φX174’, *J. Mol. Biol.* **1**(1), 37–42 (1959).

29 Vladimir Sertić and Nikolai Boulgakov, ‘Groupement des souches d’*Escherichia coli* à l'aide des lysines B sécrétées par les bactériophages’, *C. R. Soc. Biol.* **132**, 444–446 (1939); Vladimir Sertić and Nikolai Boulgakov, ‘Sur l'examen de la polyvalence des bactériophages et de leurs lysines diffusibles’, *C. R. Soc. Biol.* **132**, 442–444 (1939).

30 Milne, *op. cit*. (note 6).

31 Rachel A. Smith, Nkuchia M. M'ikanatha and Andrew F. Read, ‘Antibiotic resistance: a primer and call to action’, *Health Commun.* **30**(3), 309–314 (2015).

32 David Trudil, ‘Phage lytic enzymes: a history’, *Virol. Sin.* **30**(1), 26–32 (2015).

## Appendix: complete bibliography of Vladimir Sertić^33^

I. *Single-authored papers*

1. ‘Procédé d'obtention de variantes du bactériophage adaptées à lyser des formes bactériennes secondaires’, *C. R. Soc. Biol.* **100**, 612–614 (1929).
2. ‘Contribution à l’étude des phénomènes de variation du bactériophage’, *C. R. Soc. Biol.* **100**, 614–616 (1929).
3. ‘Origine de la lysine d'une race du bacteriophage’, *C. R. Soc. Biol.* **100**, 477–479 (1929).
4. ‘Sur des races de bactériophages virulentes pour les streptocoques’, *C. R. Soc. Biol.* **102**, 982–984 (1929).
5. ‘Application de la méthode de purification des cultures mixtes de d'Herelle, à la recherche de nouvelles races de streptophages’, *C. R. Soc. Biol.* **105**, 534-535 (1930).
6. ‘Diffusion de la lysine et pénétration des bactériophages autour de la plage’, *C. R. Soc. Biol.* **118**, 629–630 (1935).
7. ‘Agglutination microbienne et lysine du bactériophage’, *C. R. Soc. Biol.* **119**, 780–781 (1935).
8. ‘Sur l'inactivation des bactériophages par des bactéries’, *C. R. Soc. Biol.* **124**, 218–220 (1937).
9. ‘Sur l'action inhibitrice des cations monovalents sur la multiplication d'une race de bactériophage’, *C. R. Soc. Biol.* **124**, 14–15 (1937).
10. ‘Sur la différence d'action des électrolytes sur le développement des diverses races de bactériophages’, *C. R. Soc. Biol.* **124**, 98–100 (1937).
11. ‘Sur le rôle de la lysine B au cours de la bactériophagie’, *C. R. Soc. Biol.* **126**, 1074–1076 (1937).

II. *Papers co-authored with Nikolai Boulgakov*

1. ‘Lysine de bactériophages présentant différents thermo-résistances’, *C. R. Soc. Biol.* **108**, 948–950 (1931).
2. ‘Sur des bactériophages hyper virulents spécifiques pour bacillus typhi’, *C. R. Soc. Biol.* **111**, 605–606 (1932).
3. ‘Sur une méthode d’évaluation de la virulence d'un bactériophage’, *C. R. Soc. Biol.* **111**, 564–565 (1932).
4. ‘Sur des bactériophages virulents pour *Chromobacterium violaceum*’, *C. R. Soc. Biol.* **118**, 630–631 (1935).
5. ‘Isolement de multiples races de bactériophages, actifs vis-à-vis d'une bactérie donnée’, *C. R. Soc. Biol.* **119**, 492–493 (1935).
6. ‘Sur la sensibilité des souches d’*Eberthella typhi* au bactériophage, en relation avec les caractères antigéniques’, *C. R. Soc. Biol.* **122**, 35–37 (1936).
7. ‘Le virus bactériophage (*Protobius bacteriophagus*, d'Herelle 1918)’, *Médecine* **17**(suppl. 2), 6 (1936).
8, ‘Sur l'action bactéricide du bactériophage’, *C. R. Soc. Biol.* **123**, 778–779 (1936).
9. ‘L'agglutination par la trypaflavine des *Salmonella* de structure antigénique flagellaire non spécifique’, *C. R. Soc. Biol.* **123**, 951–952 (1936).
10. ‘Bactériophages spécifique pour des variétés bactériennes flagellées’, *C. R. Soc. Biol.* **123**, 887–888 (1937).
11. ‘Réversion, par acidification, de l'agglutination par la trypaflavine’, *C. R. Soc. Biol.* **124**, 217–218 (1937).
12. ‘Sur la sensibilité d'une souche d’*Escherichia coli* au bactériophage, en relation avec les caractères antigéniques’, *C. R. Soc. Biol.* **126**, 734–736 (1937).

III. *Other co-authored papers*

1. Vladimir Sertić and W.-I. Gough, ‘Modification d'une race de bactériophage par adaptation sur des formes bactériennes secondaires’, *C. R. Soc. Biol.* **105**, 199–201 (1930).
2. Pierre Giraud and Vladimir Sertić, ‘Action de hauts champs centrifuges sur divers cellules bactériennes, sur différents bactériophages et la lysine diffusible d'un bactériophage’, *C. R. Soc. Biol.* **118**, 1286–1288 (1935).
3. Felix d'Herelle and Vladimir Sertić, ‘Formation, par adaptation, de races de bactériophages thermo-résistantes’, *C. R. Soc. Biol.* **104**, 1256–1260 (1930).

